# Drug–Drug Interactions with Oral Anticoagulants as Potentially Inappropriate Medications: Prevalence and Outcomes in Elderly Patients in Primary Care and Hospital Settings

**DOI:** 10.3390/pharmaceutics14071410

**Published:** 2022-07-05

**Authors:** Mathilde Bories, Guillaume Bouzillé, Marc Cuggia, Pascal Le Corre

**Affiliations:** 1 Pôle Pharmacie, Service Hospitalo-Universitaire de Pharmacie, CHU de Rennes, 35033 Rennes, France; mathilde.bories@chu-rennes.fr; 2 CHU Rennes, INSERM, LTSI-UMR 1099, Univ Rennes, 35000 Rennes, France; guillaume.bouzille@univ-rennes1.fr (G.B.); marc.cuggia@univ-rennes1.fr (M.C.); 3 Laboratoire de Biopharmacie et Pharmacie Clinique, Faculté de Pharmacie, Université de Rennes 1, 35043 Rennes, France; 4 CHU Rennes, INSERM, EHESP, Irset (Institut de Recherche en Santé, Environnement et Travail)-UMR_S 1085, Univ Rennes, 35000 Rennes, France

**Keywords:** drug–drug interactions, combined CYP3A4–P-gp, oral anticoagulants, bleeding risk, potentially inappropriate medications, clinical data warehouse, machine learning

## Abstract

Direct oral anticoagulants and vitamin K antagonists are considered as potentially inappropriate medications (PIM) in several situations according to Beers Criteria. Drug–drug interactions (DDI) occurring specifically with these oral anticoagulants considered PIM (PIM–DDI) is an issue since it could enhance their inappropriate character and lead to adverse drug events, such as bleeding events. The aim of this study was (1) to describe the prevalence of oral anticoagulants as PIM, DDI and PIM–DDI in elderly patients in primary care and during hospitalization and (2) to evaluate their potential impact on the clinical outcomes by predicting hospitalization for bleeding events using machine learning methods. This retrospective study based on the linkage between a primary care database and a hospital data warehouse allowed us to display the oral anticoagulant treatment pathway. The prevalence of PIM was similar between primary care and hospital setting (22.9% and 20.9%), whereas the prevalence of DDI and PIM–DDI were slightly higher during hospitalization (47.2% vs. 58.9% and 19.5% vs. 23.5%). Concerning mechanisms, combined with CYP3A4–P-gp interactions as PIM–DDI, were among the most prevalent in patients with bleeding events. Although PIM, DDI and PIM–DDI did not appeared as major predictors of bleeding events, they should be considered since they are the only factors that can be optimized by pharmacist and clinicians.

## 1. Introduction

With the aging of the population and the emergence of chronic diseases requiring polymedication, the risk of exposure to drug–drug interactions (DDI) and consequently the risk of ADEs and hospitalizations is increased [1,2]. DDIs are usually defined as any co-prescription of several drugs known to interact with each other. Indeed, DDI refers to clinically significant and unintended change in the exposure and/or effect of one drug in response to the co-administration of another drug [3]. They may be of pharmacokinetic (i.e., a change in exposure) or pharmacodynamic origin (i.e., a change in effect) [4]. Using a large panel of 66 potential DDIs, it was shown that the five most common DDIs were of pharmacodynamic origin, accounting for 80% of all DDIs [5].

A review indicated that the impact of DDIs on adverse patient outcomes was uncertain, and that DDIs were linked to approximately 0.05% of ED-visits, 0.6% of hospital admissions and 0.1% of re-hospitalizations [6]. A more recent review and meta-analysis indicated that DDIs were a significant cause (22.2%, interquartile range 16.6–36.0%) of hospital admissions and hospital visits [2]. Furthermore, this study highlighted that NSAIDS were the drugs most involved in DDI-related hospital admission, and that DDI-related hospital visits (outpatient and emergency department) involved warfarin [2]. A prospective study carried out in France (EMIR study) showed that 3.6% of hospitalizations were related to an ADE and that DDIs accounted for 30% of the hospitalizations [7]. Furthermore, vitamin-K antagonists were the drugs most frequently associated with hospital admission. A study evaluating the prevalence of potentially clinically significant DDIs after hospital admission indicated that the prevalence of DDIs was high (54%) at hospital admission, increased during hospitalization with DDIs in older patients and remained stable over 12 months [5]. This study showed that drug interactions involving an oral anticoagulant (vitamin-K antagonists or direct thrombin inhibitor) with an antiplatelet or NSAIDs drugs were ranked in the top five DDIs.

Within elderly people, recent studies have found an increase in the prevalence of ADE-related hospitalizations with a rate of hospitalization of 8.7% in people older than 60 years old and of 10% in those older than 65 years, respectively [8,9]. Several lists of drugs considered as potentially inappropriate medications (PIM) in the elderly have been proposed. These PIM drugs represent any drug suspected of having an unfavorable bene-fit/risk ratio and/or limited efficacy compared to other therapeutic alternatives in the elderly [10]. Among the many tools developed for detecting these PIMs, the American Geri-atrics Society’s Beers Criteria are the most used [10].

It should be noticed that PIM criteria (including more than 90 drugs on the Beers list) are regularly updated but involve only a very limited number of DDIs. However, DDI occurring specifically with PIM (i.e., PIM–DDI) seems a relevant issue, although we showed it was poorly studied [11]. Indeed, it could be hypothesized that the inappropriate feature of these PIM drugs (i.e., unfavorable benefit/risk ratio) could be increased by a drug–drug interaction since for most the drugs ADRs are related to the systemic concentrations. For example, an interaction by a metabolic or a transporter inhibition could increase the systemic exposure of the PIM and thus may increase the frequency and/or severity of ADEs. In a descriptive study of pharmacokinetic DDI involving PIM we showed a prevalence ranging from 0.10% (Tramadol–Terbinafine) to 19.04% (Apixaban–Amiodarone) [11]. These elements highlight the fact that DDI involving PIM (PIM–DDI) should be considered and the potential clinical outcomes of these PIM–DDI should be questioned. 

Investigating the clinical consequences of PIM–DDI involving oral anticoagulants, drugs ranked in the top four of the drug classes at hospital admission in elderly patients should be of value [8]. 

Oral anticoagulants (VKAs and DOACs) are considered PIMs in elderly patients in several specific situations (patient age ≥ 75 years, patient in renal failure [10]), and the main ADE highlighted by the Beers criteria is bleeding risk with notably increased risks of gastrointestinal or cerebral hemorrhage. 

Subject to numerous pharmacokinetic, pharmacodynamic and dietary interactions, it has been shown that interactions with anticoagulants were responsible for 56.7% of hemorrhagic ADEs for new oral anticoagulants (NACOs) [12] and 61% of hemorrhagic ADEs for warfarin [13]. In addition, the number of DDIs appeared to be significantly associated with the risk of bleeding [12]. 

This information highlights the complexity of DDI and oral anticoagulants, and the influence of pathophysiological conditions on the magnitude of the DDI. Since oral anticoagulants are considered PIM in specific situations in elderly patients, the impact of PIM–DDI on the bleeding outcomes deserve to be investigated, which has not yet been performed. Furthermore, the development of a tool to predict the bleeding risks associated with these PIM–DDI also seems of value to improve patient management. Indeed, there is a need to better understand the predictors of ADE in elderly people, and the inter-relations between the different predictors [14]. 

The aim of the study was to perform a descriptive analysis of the prevalence of PIM, DDI and PIM–DDI in patients in primary care setting and during hospitalization and to evaluate their link with bleeding events in elderly patients. A secondary aim of the study was to develop an ADE prediction tool of bleeding risk for elderly people treated with oral anticoagulants and to rank specific PIM–DDI within the predictors.

## 2. Materials and Methods

We conducted a retrospective cohort study in elderly patients hospitalized and treated with oral anticoagulants. The study is based on the dataset used by the INSHARE (Integrating and Sharing Health Big Data for Research) project [15]. We initiated the INSHARE project to allow the link between primary care clinical data and hospital clinical data in order to analyze the care trajectories of patients and particularly their therapeutic pathway. Therefore, a semideterministic record linkage method based on the common variables between primary care and hospital data sources was developed. Common variables allowing the link between the two databases were PMSI (Programme de médicalisation des systèmes d’information) variables, which are available and produced in a standardized way for all French hospitals. Hence, this link allowed us to obtain all the drug treatment of the patients in primary care before hospitalization. Further technical information on the linkage procedure used to create the INSHARE patient cohorts, as well as a case use, has been published [15].

### 2.1. Data Sources

The SNDS primary care database (Système National des Données de Santé) is the national repository of medico-administrative data covering 98.8% of the French population [16]. It contains data on outpatient care, such as physician consultation, drug dispensing by community pharmacies and data on inpatient care as well as disease diagnosis and medical procedures received by the patient during hospital stay. For each patient, drug dispensing by community pharmacies in a primary care setting and socio-demographic characteristics allowing matching data from the hospital data warehouse (i.e., month and year of birth; gender; PMSI information on hospital stays, such as disease diagnosis, date of admission, date of discharge, length of stay, etc.) were collected.

eHOP is the hospital clinical data warehouse (CDW) we developed, and it is used at the University Hospital of Rennes [17]. It contains the administrative and clinical data of each patient hospitalized or in outpatient consultation at the hospital, such as date of admission and discharge, drug administration or medical biology analyses. Overall, eHOP covers more than 1.4 million patients. Socio-demographic characteristics (i.e., month and year of birth, gender and PMSI information on hospital stays, e.g., diagnosis, date of admission, date of discharge, length of stay) allowed matching with the SNDS database. Drug administrations (date of administration, ATC classification), laboratory data (INR, hemoglobin) and information on patient stay (date of admission, date of discharge, length of stay, ICD-10 diagnosis codes) were collected for each patient.

The extracted data did not contain any nominative data, all information has been de-identified in accordance with the protocol established for the INSHARE project [15]. The agreement of the French Commission Nationale de l’Informatique et des Libertés (CNIL) was obtained (CNIL Agreement N° 2206739).

### 2.2. Study Population and Follow-Up

Patients hospitalized at the University Hospital of Rennes from 1 January 2015 to 31 December 2017, were ≥65 years old and to whom oral anticoagulants were dispensed in primary care (the month prior hospitalization) and/or administered during hospital were included. Patients only hospitalized for an infra-day period were not included in the INSHARE project (e.g., anticancer chemotherapy, dialysis, apheresis, blood transfusion and hyperbaric oxygen therapy). 

For each patient, data from SNDS primary care database were extracted over a period ranging from 1 year before the first stay at Rennes University Hospital. This period allowed us to detect previous hospitalization in another hospital due to bleeding events, to compute the Charlson score and to estimate the duration of exposure to oral anticoagulants. This allowed us to characterize the anticoagulant therapeutic pathway resulting from potential modification in oral anticoagulant treatment (end and/or initiation).

### 2.3. Exposure Assessment

#### 2.3.1. Oral Anticoagulant Exposure

The identification of oral anticoagulants considered as PIM was based on the Beers criteria [10] are described as follows:

- Apixaban: Patient ≥ 65 years old with renal impairment
- Dabigatran: Patient ≥ 75 years old or patient ≥ 65 years old with renal impairment
- Rivaroxaban: Patient ≥ 75 years old or patient ≥ 65 years old with renal impairment
- Warfarin: Patient ≥ 65 years old with the following drug combinations:
  o Warfarin–Amiodarone,
  o Warfarin–Sulfamethoxazole/Trimethoprim,
  o Warfarin–Ciprofloxacin,
  o Warfarin–Macrolides (excepted Azithromycin)
  o Warfarin–Non-steroidal anti-inflammatory drugs (NSAIDs)

The detection of treatment by oral anticoagulants identified as PIM was based on the ATC classification (apixaban B01AF02, dabigatran B01AE07, rivaroxaban B01AF01 and warfarin B01AA03). 

#### 2.3.2. DDI and PIM–DDI Exposure 

DDI interactions were checked by using Theriaque drug database [18], focusing on DDI with bleeding risk. Given the differences reported in the literature in the detection of DDIs, DDI of highest risk (contra-indication and major) from the Micromedex database [19] were added to those reported in Theriaque.

All DDI and specific PIM–DDI were recorded in all the patients. For each DDI or PIM–DDI, the type of interaction (pharmacokinetic or pharmacodynamic), mechanism of action and level of severity (1: contraindicated, 2: not recommended, 3: use with caution and 4: to take into account) were considered. Given the fact that there are variations in the classification of DDI and PIM–DDI among Theriaque and Micromedex, we used a mapping of the DDI risk classification based on the descriptions given by the providers [11]. As a result, we considered that major DDI/PIM–DDI refers to levels 1 and 2 of Theriaque (“contraindicated” and “not recommended”) and to the “contraindicated” and “major” levels of Micromedex. Moderate DDI/PIM–DDI refers to levels 3 of Theriaque (“use with caution”) and to the “moderate” level of Micromedex. Level 4 of Theriaque (“to take into account”) was classified as a minor DDI/PIM–DDI.

The identification of patients with PIM–DDI involving oral anticoagulants was made possible by retrieving the variables indicated in the Beers criteria (abovementioned).

DDI and PIM–DDI were detected in the pre-hospitalization period (30-day period) and during the hospital stay.

### 2.4. Detection of Patients with Bleeding ADE

The detection of patients with a hospital admission for bleeding ADE within 30 days after oral anticoagulation dispensing in primary care setting was based on the presence of one or more of the ICD-10 diagnostic codes provided in Table S1. This list was established using several sources (ICD-10 codes for bleeding ADEs of oral anticoagulants provided by the Theriaque database and the bleeding ADEs provided by the SIDER database) [20]. A bleeding ADE was also defined with an INR > 5 or a decrease in hemoglobin of at least 2 g/dL compared with the minimum usual values (i.e., a hemoglobin less than or equal to 11 g/dL for men and 10 g/dL for women [21]. The last method used to detect ADEs was the administration of a known antidote to oral anticoagulants: vitamin K, idarucizumab, andexanet alpha or human prothrombin complex during hospitalization, based on ATC classes (ATC classes: B02BA02, V03AB37, V03AB38, B02BD01).

### 2.5. Descriptive Analyses

Prevalence of PIM, DDI, PIM–DDI and bleeding ADE were determined. Patients anticoagulant drug pathway were described (interruption or initiation of oral anticoagulant treatment, as well as the evolution of DDI or PIM–DDI prevalence before and during hospitalization).

### 2.6. Exploratory Analyses

Patients included in these exploratory analyses were those who received an oral anticoagulant in primary care setting within 30 days before their hospital stay.

The association between known predictive variables (age, sex, presence of PIM, history of stroke, previous bleeding event, diabetes, renal disease, liver disease, cancer and hypertension) [22,23], potential predictive variables (presence of DDI, presence of PIM, presence of PIM–DDI) and the variable to be predicted (bleeding ADE) was measured by logistic regression where the odds ratios and associated 95% confidence interval of each variable were calculated.

The search for other ADEs’ predictive variables was based on several supervised learning method: random forest (RF), support vector machine (SVM) and extreme gradient propulsion (XGBoost). As the dependent variable was a binary variable (presence or absence of bleeding ADE), algorithms developed were of classification-type. RF and XGBoost techniques adopted the tree-based modeling algorithm, which is a tree model capable of synthesizing the analysis to get the best prediction decision. The SVM algorithm consisted in reducing a classification or discrimination problem to a hyperplane, in which the data are separated into several classes using a limit maximizing the distance between the different groups of data. The importance of variables was sorted via the XGBoost algorithm, the importance of a variable corresponds to the performance gain of the algorithm each time the variable is used. Thus, the more a variable is used the higher its global gain will be.

The evaluation of the models followed a classical learning methodology with a training sample comprising 80% of the patients, with the remaining 20% as test sample (sampling was stratified on ADE to have equal proportions of ADE in the training set and in the test set). 

For each model, a workflow was performed, including a class rebalancing step using SMOTE method (synthetic minority oversampling technique). Each model was 10-fold cross-validated using the training sample in order to fine tune hyperparameters. The receiver operating characteristic (ROC) curve on the training sample was used to optimize hyperparameters. Accuracy, sensitivity and specificity were computed at the threshold giving the minimum Euclidean distance between the (specificity and sensitivity) coordinates and the optimal point (1,1). 

To evaluate performance, the area under the curve (AUC) in the ROC curve was performed on the test set for the best model of each algorithm family. The R software (v. 3.6.0) and the “tidymodels” package [24] were used to develop and evaluate the machine learning-based models.

## 3. Results

### 3.1. Descriptive Analyses

#### 3.1.1. General Characteristics and Patient Drug Pathway

Out of the 159,485 patients available in the INSHARE cohort, 3.5% patients (*n* = 5583) patients were ≥65 years old and had received oral anticoagulant therapy the month before hospitalization (2.4%, *n* = 3867) or during hospitalization (2.2%, *n* = 3595). The general characteristics of the patient and of their anticoagulant treatments are presented in Table 1. 

**Figure 1:**
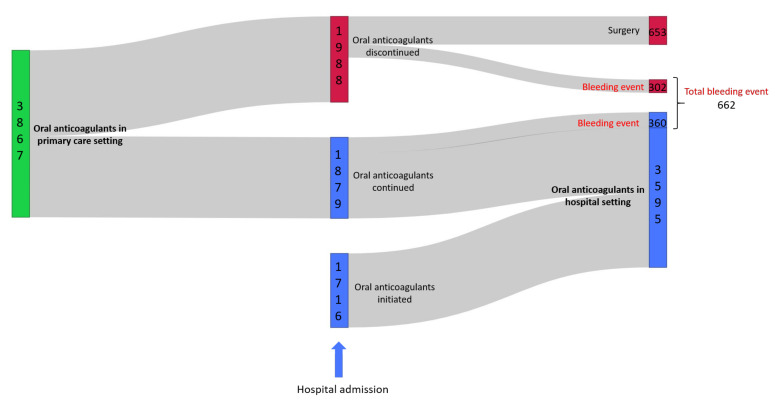
Sankey diagram representing the oral anticoagulant pathway of the 5583 hospitalized patients treated by oral anticoagulants in primary care and/or during their hospitalization.

The anticoagulant treatment pathway of the cohort of patients is illustrated in Figure 1. Of the 3867 patients with oral anticoagulation prior to hospitalization, 48.6% (*n* = 1879) continued their anticoagulant therapy during their hospital stay while oral anticoagulants treatment was discontinued in 51.4% patients (*n* = 1988). Within patients in whom oral anticoagulants treatment was stopped, a bleeding event was noticed in 15.2% (*n* = 302) patients and 32.8% (*n* = 653) were hospitalized for surgical procedure. For the remaining 52.0% (*n* = 1033) patients, the discontinuation of the anticoagulation could be explained by a short hospital stay that did not lead to a hospital prescription, by an inadvertent discontinuation in anticoagulant treatment that was not detected because of the absence in medication reconciliation, by a switch in oral anticoagulant oral anticoagulant to parenteral heparins or by an untracked medication administration. However, within patients in whom oral anticoagulants were continued, a bleeding event was noticed in 19.2% patients (*n* = 360). The initiations of oral anticoagulant therapy during hospitalization were achieved in 47.8% (*n* = 1716) of the patients.

#### 3.1.2. Prevalence of PIM, DDI and of PIM–DDI

Within anticoagulant treatments, the prevalence of PIM, DDI and PIM–DDI (Table 2) were not very different between primary and hospital settings. In each setting, the prevalence of DDI with warfarin was higher than for NOACs with the following the rank order: apixaban > rivaroxaban > dabigatran. The prevalence of PIM–DDI with warfarin was close to that of NOACs with the following rank order: rivaroxaban > dabigatran > apixaban. The average number of DDI per patient was 2-fold higher than that of PIM–DDI without a difference between settings. The contra-indicated DDI and PIM–DDI were around 1.5-fold higher in hospital settings.

**Figure 2:**
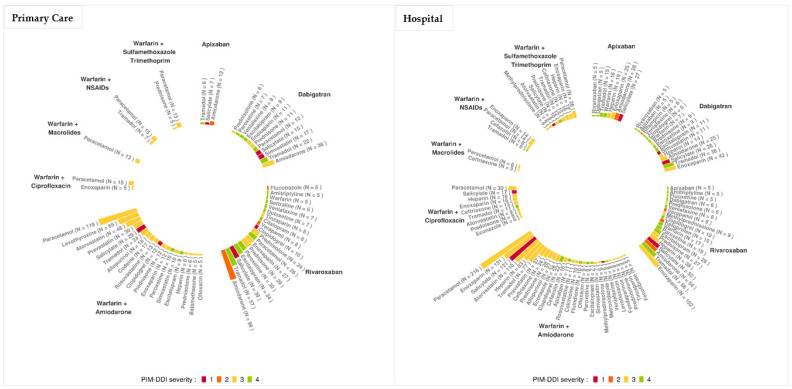
Ranking of the drugs involved in PIM–DDI in hospitalized patients treated by oral anticoagulants in primary care and/or during their hospitalization. Levels of severity: Contraindicated (1, red), not recommended (2, orange), use with caution (3, yellow) and to take into account (4, green).

The drugs most frequently involved in PIM–DDI in patients (occurring in more than five patients) with oral anticoagulant therapy in primary care setting and during hospitalization and their levels of severity are presented in Figure 2 (Primary Care) and Figure 2 (Hospital). This figure clearly shows that warfarin and rivaroxaban are the anticoagulants most involved in PIM–DDI with a wide variety of drugs.

In primary care settings, paracetamol associated with warfarin–amiodarone was the most frequent PIM–DDI (*n* = 119 patients). However, this combination was of low severity (3: use with caution). The most severe PIM–DDI reported was level 2 severity (not recommended), associating rivaroxaban and amiodarone (*n* = 96 patients).

In hospital settings, warfarin–amiodarone plus paracetamol was also the most frequent PIM–DDI association reported (*n* = 218 patients). The most severe PIM–DDI was of level 1 (contraindicated) associating warfarin–amiodarone and salicylate (*n* = 121 patients).

#### 3.1.3. Mechanisms of DDI and of PIM–DDI

Table 3 indicates that the main mechanisms of DDI in primary care setting are of pharmacokinetic origin (61.3%), whereas DDI of pharmacodynamic origin were the most frequent during hospitalization (59.8%) as a result of the shift from DOAC to heparin (i.e., the temporary co-prescription of heparins and DOAC during stay). Combined CYP3A4 and P-gp inhibition accounted for 24.5% (*n* =197 patients) of pharmacokinetic DDI in primary care and 22.3% (*n* = 223 patients) during hospitalization. 

Concerning PIM–DDI mechanisms, similarly to DDI mechanisms, the PIM–DDI of pharmacokinetic origin were the most frequent in primary care settings (71.1%) as opposed to hospital settings where PIM–DDI of pharmacodynamic origin were the most represented (54.9%). Combined CYP3A4 and P-gp inhibition accounted for 21.4% (*n* = 116 patients) in primary care and 18.2% (*n* = 88 patients) in hospital settings. 

Combined CYP3A4 and P-gp inhibition was reported with apixaban and rivaroxaban and was due to amiodarone, verapamil, diltiazem, ciclosporin or dronedarone.

#### 3.1.4. Bleeding ADE at Admission

There were 17.1% (*n* = 662, Figure 1) patients treated with oral anticoagulants in primary care setting who were hospitalized for bleeding ADE. Most frequent ICD-10 diagnoses of bleeding ADEs were: occurrence of adverse effects of anticoagulants during their therapeutic use (*n* = 105 patients), hematuria (*n* = 62 patients), acute post hemorrhagic anemia excluding anemia due to chronic blood loss, iron deficiency anemia or congenital anemia from fetal blood loss (*n* = 58 patients), recto-anal hemorrhage (*n* = 42 patients), melena (*n* = 30 patients) and epistaxis (*n* = 30 patients). 

The diagnosis of bleeding involving an abnormal laboratory result, such as INR > 5 or hemoglobin level well below the usual values, concerned 58.7% (*n* = 389) patients. Finally, 3.8% (*n* = 25) patients were identified as having had a bleeding ADE by the of an antidote specific to the anticoagulants studied. 

Of the 662 patients involved, 54.5% (*n* = 361 patients) were treated with warfarin and 45.9% (*n* = 304 patients) with direct oral anticoagulants. 

Among patients with either PIM, DDI or PIM–DDI in primary care setting, the rate of hospitalization for bleeding ADE was 19.4% (*n* = 172 patients) of patients with PIM, 17.3% of patients with DDI (*n*= 316 patients) and 20.3% (*n* = 153 patients) of patients with PIM–DDI. 

The top 10 DDIs and PIM–DDIs and their severity level in primary care patients with a bleeding ADE are reported in Table 4.

### 3.2. Exploratory Analyses

#### 3.2.1. Logistic Regression 

The result of the logistic regression presented in Table 5 indicates that age, history of bleeding event, renal disease, cancer and hypertension can be considered as variables associated with hospitalization for bleeding events. PIM and DDI were not evidenced as risk factors but PIM–DDI were close to significance (OR: 1.23; 95% CI: 1.00–1.57, *p* = 0.060).

#### 3.2.2. Machine Learning to Predict Hospitalization for Bleeding Event

The RF, XGBoost and SVM models were developed to predict hospitalization due to bleeding events. 

**Figure 3:**
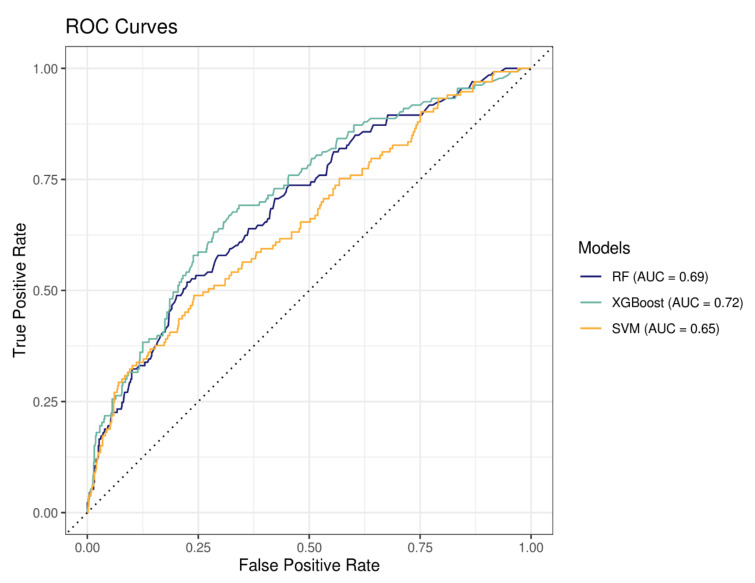
Receiver operating characteristic (ROC) curves representing the sensitivity (true positive rate) as a function of 1-specificity (false positive rate) for all possible thresholds values. RF = random forest, XGBoost = extreme gradient propulsion, SVM = support vector machine and AUC = area under the curve.

For each model, training samples contained 3093 patients and testing samples 774. Of these, 17.2% in each sample were hospitalized for bleeding events (*n* = 529 patients in the training sample and *n* = 133 patients in the testing sample). Performances of the three models presented in Figure 3 and Table 6 indicated that the XGBoost model had the better performance (highest ROC AUC = 0.72). 

**Figure 4:**
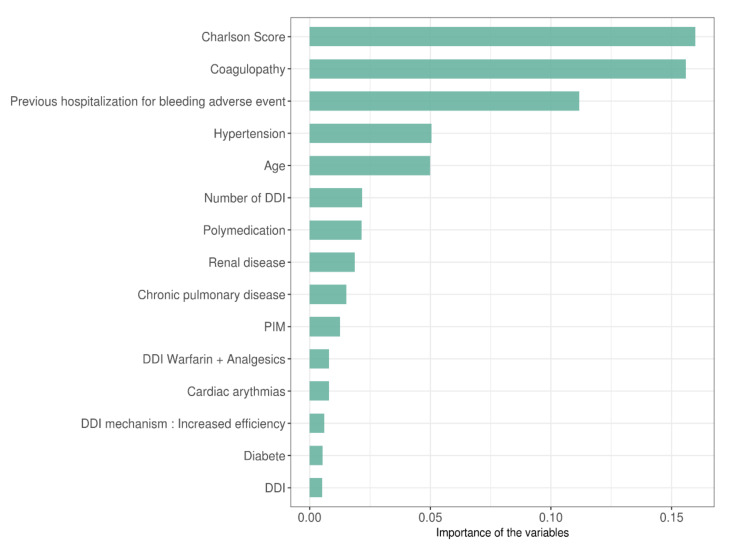
Importance of the variables selected by XGBoost algorithm ranked by importance scores.

The importance of variables (ranking the 15 most important) explaining bleeding events obtained by the XGBoost algorithm are shown in Figure 4. The number of DDI involving anticoagulants per patient, the polymedication, the presence or not of a PIM and the presence or not of a DDI were ranked 6th, 7th, 10th, and 15th, respectively. PIM–DDI was not ranked in the top 15 but was ranked in the 20th position over 50 variables.

## 4. Discussion

Concerning general characteristics, 70% of the patients were aged ≥ 75 years, mostly hospitalized for medical (i.e., non-surgical) conditions and treated with oral anticoagulants for more than 6 months. Warfarin was the most prescribed anticoagulant before and during hospitalization in our sample of patients. Despite the general rise in prescriptions of DOACs in elderly patients, warfarin is still widely used today [23]. Indeed, it has been pointed out that for patients ≥ 75 years old, warfarin had an equivalent safety compared to DOACs, even in patients with impaired renal function [23].

The linkage between ambulatory database (SNDS) and hospital data warehouse (eHOP) allowed us to display the anticoagulant treatment pathway (Figure 1), showing that for 51.4% of the patients the oral anticoagulation was discontinued. Although for some patients the reasons for discontinuation were clear (surgery, ADE bleeding), other patients had no apparent reason for discontinuation at their admission to the hospital, the transition between settings should be further monitored to avoid this type of treatment disruption. This also showed that, in a significant proportion of patients, the oral anticoagulant treatment was continued even though a bleeding event was reported. However, ICD-10 diagnosis indicated that these bleeding events were of minor severity.

We previously showed in a systematic review that the total prevalence of PIM (i.e., based on all therapeutic classes) was significantly higher in hospital setting than in primary care setting (44.6% vs. 19.1%) [11]. However, in the current study, focusing only on oral anticoagulants, the prevalence of PIM was lower and similar before and during hospitalization (22.9% vs. 20.9%). This difference should be explained by the well-known risk associated with this class of drugs, leading to a higher vigilance of health care professionals in hospital setting. This may suggest that the concept of PIM per se should not be of utmost significance for therapeutic classes and/or drugs leading to high risk ADE. However, the prevalence of PIM within oral anticoagulants is far from negligible and the way they could be reduced should be questioned, as well as their potential impact on bleeding ADE.

The prevalence of DDI and of PIM–DDI of all severity levels was slightly higher during hospitalization than before hospitalization (Table 2), as well as their prevalence of major severity. These results are in agreement with those obtained in previous studies aimed at comparing primary care and hospital DDI prevalence and displaying a higher average prevalence during hospitalization [11,15]; however, the difference we showed was less marked for oral anticoagulants. Although we demonstrated marked differences in both DDI and PIM–DDI between warfarin and DOACs, warfarin is still the drug showing the highest prevalence of drug interactions, considering both DDI and PIM–DDI.

Within DDI and PIM–DDI, the inhibition of CYP2C9 and of CYP2C19 was the most prevalent drug interaction, involving mainly escitalopram, paroxetine or citalopram with warfarin or rivaroxaban. However, the current study showed that the combined inhibition of CYP3A4 and P-gp was quite as frequent and much more frequent than the sole inhibition of CYP3A4. CYP3A4 and P-gp share not only overlapping tissue distribution (especially at the intestine level) and substrate specificity but also share dual inhibitors. Hence, drugs that are both substrates of these biological systems may undergo significant DDI if a perpetrator is a dual inhibitor (e.g., itraconazole, antiviral protease inhibitors and cardio-vascular drugs, such as amiodarone, verapamil and diltiazem). Indeed, the interplay between enzyme and transport systems can lead to DDI of high intensity since the inhibition of intestinal efflux transporters decreases drug cycling between the enterocytes and gut lumen so that more drug molecules escape the CYP3A4 metabolism inside the enterocytes [25].

The association of amiodarone and rivaroxaban (level 2 severity DDI) as a prototypic combined inhibition of CYP3A4 and P-gp was a quite frequent DDI in the primary care setting (9.1%, *n* = 42 patients) and much more prevalent as a PIM–DDI in frail patients with impaired renal function (37.8%, *n* = 96 patients). A recent case–control retrospective study has shown that DDIs involving DOACs were linked to an increased risk of bleeding when associated with combined P-gp/CYP3A4 inhibitors and were linked to a higher risk for stroke and systemic emboli when associated with CYP3A4 inducers [26]. In this study, an increased risk of serious bleeding was reported when associating rivaroxaban with amiodarone (OR = 1.68, 95 CI = 1.14–2.49 for amiodarone) [26].

In our study, this association (amiodarone and rivaroxaban), as a PIM–DDI, was reported in 18.8% of the patients with a bleeding event. These results are consistent with a recent systematic review highlighting that DDI-related bleeding events preferentially resulted from a simultaneous inhibition of CYP3A4 and P-gp [27]; even though pharmacokinetic DDI involving DOACs were not many. It should also be noticed that amiodarone, as a CYP2C9 inhibitor, has been associated with over-anticoagulation in patients treated with warfarin [28] and the variability of the magnitude of the warfarin–amiodarone DDI is associated with the renal function [29].

In our study, the main risk factors for bleeding were mainly of pathophysiological origin (Table 5) and PIM, DDI and PIM–DDI per se were not risk factors of bleeding. However, the odds ratio for PIM–DDI was close to statistical significance, suggesting that the evaluation of PIM–DDI as a risk factor of bleeding should be carried out in a larger cohort of patients.

The second aim of this study was then to identify the variables capable of predicting general bleeding events and to quantify the impact of DDI, PIM or PIM–DDI on the occurrence of such events by using machine learning (ML) methods.

Several bleeding prediction tools have been developed in the past years, such as HEMORR2HAGES [30], HAS-BLED [31] or ATRIA scores [32]. However, these predictive tools have been evaluated in very specific contexts like atrial fibrillation (HAS-BLED score) or the initiation of vitamin K antagonist therapy (HEMORR2HAGES). Moreover, if HAS-BLED [31] mentions drug interactions in its bleeding predictors, none of the other bleeding scores refer to potential prescribing determinants, such as DDI, PIM or PIM–DDI. Given the fact that the performance of prediction depends on the data source and methods used, we hypothesized that ML methods applied to a large and recent patient dataset with a wide range of variables could produce a risk model with superior performance compared to these existing scores. Indeed, ML methods have already been used in a few studies to predict vascular events, such as bleeding events [33,34]. However, once again, these studies were focused on specific contexts and did not consider the prescribing determinants (DDI, PIM, and PIM–DDI) in their analysis.

Among the different ML algorithms we used, the XGBoost model showed the highest specificity in discriminating patients with and without a bleeding event (AUC = 0.72) and identified 70% of patients with a bleeding event, which is the highest sensibility among the models tested in this study.

Concerning the predictive variables we identified, most of them were included by HEM-ORR2HAGES [30], HAS-BLED [31] or ATRIA [32] scores, such as hypertension, age or previous bleeding events. Similarly, kidney disease, chronic pulmonary disease and diabetes have also been also identified as relevant by Claxton et al. [33] in a previous study.

Our study highlights that the development of machine learning algorithms could improve care for patients treated with oral anticoagulants considered to be PIMs. Beyond this, leveraging retrospective analytics from CDW big data should help the development of predictive tools to predict and prevent adverse events, such as hospitalization, hospital readmission and the stratification of patients with a high risk of drug-related adverse events [35].

Our analyses identified polymedication, number of DDI, presence of PIM and presence of DDI as predictors of hospitalization for bleeding events. Even though they are not all ranked the highest, they should be considered because these variables can be influenced by an optimization of the drug treatment (i.e., deprescription, lower prescription of PIM and better vigilance towards DDI).

## 5. Strengths and Limitations

This study, focusing on hospitalization for bleeding events due to oral anticoagulants and, more specifically, to their link with PIM, DDI and DDI involving PIM (i.e., PIM–DDI), used data on drug use and on biological data in patients both before their hospitalization and during their hospital stay.

The integration of the ambulatory database (SNDS database) with the hospital data warehouse (eHOP) allowed us to describe the use of oral anticoagulants in each patient hospitalized at the University Hospital of Rennes and thus to describe the prevalence of PIM–DDI throughout the patient’s care pathway (e.g., prevalence of PIM, DDI and of PIM–DDI on admission to the hospital and during the hospitalization). It also enabled us to gain access to the different patients’ hospitalization events (e.g., number of stays and origins of previous hospitalizations) but also to more precisely detect bleeding ADE by combining ICD-10 diagnostic codes and results of biological assays that are not available via the SNDS.

The analyses could have been improved by integrating data from primary care medical analysis laboratories as well as the dosing regimen of drugs delivered in primary care. Indeed, in the context of oral anticoagulant treatments and particularly of VKAs (warfarin), dosing regimen were adapted according to several patient-dependent factors, such as biological dosages (adaptation according to the INR), treatment indication or even comorbidities (e.g., renal failure).

The other important aspect of the re-use of these data was the size of the population in each group. Indeed, the literature review on PIM–DDI [11] revealed that among the studies conducted in hospitals, the population sizes studied were rather small compared to studies conducted in primary care or in nursing homes. In this case, more than 5000 patients were analyzed for their prevalence of PIM, DDI and PIM–DDI. However, despite this large study cohort, the occurrence of a bleeding ADE in patients with a PIM–DDI prescription remained a rare event, as only 153 patients were identified as such. The extension of this study on a larger scale would address this issue.

## 6. Conclusions

The current paper presents an analysis of the prevalence and impact of DDI with oral anticoagulants as PIM in elderly in both primary care and hospital settings. Linking a primary care database with a clinical hospital database allowed us to evaluate the therapeutic pathway of patients and the impact of drug treatment on their clinical outcome. Even though PIM, DDI and PIM–DDI per se were not characterized as significant risk factors of bleeding, this study showed that combined CYP3A4–P-gp drug–drug interactions as PIM–DDI are prevalent in patients with bleeding events. Although polymedication prevalence of PIM, DDI and PIM–DDI were not ranked high as predictors of bleeding risk, they should be considered since they are the only factors that can be modified, the other predictors being of pathophysiological origin. As a whole, the optimization of drug prescription in elderly patients with complex multi-morbidities is not a simple task, and it has been suggested that an “individualized, interactive, multidisciplinary, and multifaceted approach to geriatric pharmacotherapy should be promoted and encouraged” [36]. Regarding anticoagulants, stewardship programs, as already set up for antibiotics, may help in identifying high-risk prescriptions in patients to obtain a personalized anticoagulant strategy [37].

## Figures and Tables

**Table 1:** General characteristics of the hospitalized patients in both settings (primary care and hospital setting).

	Oral Anticoagulant in Primary Care Setting	Oral Anticoagulant in Hospital Setting
Total	***n* = 3867**	***n* = 3595**
**Sex**		
*Men*	57.8% (2236)	52.6% (1890)
*Women*	42.2% (1631)	47.4% (1705)
**Age (median-IQR)**	79 (73–85)	80 (73–86)
*65–75 years old*	29.5% (1141)	30.4% (1092)
*≥75 years old*	70.5% (2726)	69.6% (2503)
**Hospital stay**		
*Medicine*	63.6% (2458)	62.0% (2227)
*Surgery*	36.4% (1409)	38.0% (1368)
**Time frame exposure to oral anticoagulants prior to hospitalization**		
*≤1 month*	6.5% (253)	-
*1 to ≤3 months*	9.3% (361)	-
*3 to ≤6 months*	8.2% (318)	-
*>6 months*	75.9% (2935)	-
**Time frame exposure to oral anticoagulants during hospitalization**		
*≤1 day*	-	31.8% (1144)
*1 to ≤8 days*	-	49.0% (1763)
*8 to ≤15 days*	-	12.5% (451)
*>15 days*	-	6.6% (237)

**Table 2:** Oral anticoagulants treatments of the hospitalized patients in both settings (primary care and hospital setting). Prevalence of DDI, PIM and PIM–DDI correspond to the ratio of patients with DDI, PIM or PIM–DDI on the total of patients (*n* = 3867 in primary care and *n* = 3595 in hospital setting).

	Oral Anticoagulant in Primary Care Setting	Oral Anticoagulant in Hospital Setting
**Total**	***n*** **= 3867**	***n*** **= 3595**
**Anticoagulants**		
*Apixaban*	18.3% (706)	24.8% (890)
*Dabigatran*	11.6% (450)	7.3% (264)
*Rivaroxaban*	23.9% (923)	19.2% (690)
*Warfarine*	46.6% (1801)	50.8% (1826)
**PIM**	**22.9% (886)**	**20.9% (750)**
*Apixaban*	0.8% (33)	0.9% (31)
*Dabigatran*	4.5% (175)	3.0% (108)
*Rivaroxaban*	9.7% (374)	5.5% (199)
*Warfarin*	7.9% (304)	11.6% (417)
**DDI**	**47.2% (1825)**	**58.9% (2117)**
*Apixaban*	8.5% (329)	16.5% (593)
*Dabigatran*	2.4% (94)	1.5% (55)
*Rivaroxaban*	3.6% (140)	6.6% (239)
*Warfarin*	32.7% (1266)	34.9% (1254)
*Average number of DDI per patient*	1.5 +/− 1.8	1.6 +/− 1.8
*Contraindicated DDI (level 1)*	8.6% (243)	13.8% (607)
*Not recommended DDI (level 2)*	15.9% (450)	19.9%(871)
**PIM–DDI**	**19.5% (753)**	**23.5% (847)**
*Apixaban*	0.9% (34)	1.9% (71)
*Dabigatran*	3.6% (138)	3.6% (130)
*Rivaroxaban*	8.0% (310)	7.6% (274)
*Warfarine*	7.2% (280)	11.8% (423)
*Average number of PIM–DDI per patient*	0.8 +/− 1.9	0.8 +/− 2.0
*Contraindicated PIM–DDI (level 1)*	9.8% (116)	14.9% (266)
*Not recommended PIM–DDI (level 2)*	20.0% (236)	19.2% (344)

**Table 3:** Main mechanisms of DDI (A) and PIM–DDI (B) and their prevalence in hospitalized patients treated by oral anticoagulants in primary care and/or during their hospitalization. Combined inhibition CYP3A4 and P-glycoprotein refers to inhibition induced by amiodarone, verapamil, diltiazem, ciclosporin or dronedarone on apixaban and rivaroxaban.

A	Primary Care Prevalence of DDI (*n*)	Hospital Prevalence of DDI (*n*)
Pharmacodynamics	38.7% (507)	59.8% (1489)
Pharmacokinetics	61.3% (803)	40.2% (1001)
● Inhibition of CYP2C9 and CYP2C19	32.0% (257)	25.5% (255)
● Inhibition of CYP3A4	0.9% (7)	0.6% (6)
● Inhibition of P-glycoprotein	5.4% (43)	1.9% (19)
● Combined inhibition CYP3A4 and P-glycoprotein	24.5% (197)	22.3% (223)
**B**	**Primary care** **Prevalence of PIM–DDI** (***n***)	**Hospital** **Prevalence of PIM–DDI** (***n***)
Pharmacodynamics	28.9% (220)	54.9% (588)
Pharmacokinetics	71.1% (541)	45.1% (483)
● Inhibition of CYP2C9 and CYP2C19	26.1% (141)	23.0% (111)
● Inhibition of CYP3A4	1.5% (8)	1.4% (7)
● Inhibition of P-glycoprotein	10.0% (54)	7.9% (38)
● Combined inhibition CYP3A4 and P-glycoprotein	21.4% (116)	18.2% (88)

**Table 4:** Top 10 drugs involved in DDI or in PIM–DDI. The percentage are calculated as the ratio of the number patients with the drug combination and a bleeding ADE and the total number of patients with the drug combination.

Association	% of Patient with the Drug Combination and Bleeding ADE (*n*)	Level of Severity	Type of Association
Rivaroxaban–Salicylate	28.2% (11)	1	PIM–DDI
Warfarin–Salicylate	25.8% (33)	1	DDI
Rivaroxaban–Amiodarone	18.8% (18)	2	PIM–DDI
Warfarin–Amiodarone–Paracetamol	23.5% (28)	3	PIM–DDI
Warfarin–Paracetamol	22.6% (176)	3	DDI
Warfarin–Amiodarone–Atorvastatin	20.8% (10)	3	PIM–DDI
Warfarin–Atorvastatin	19.5% (42)	3	DDI
Warfarin–Levothyroxin	17.0% (26)	3	DDI
Warfarin–Tramadol	28.1% (34)	4	DDI
Rivaroxaban–Tramadol	22.8% (13)	4	PIM–DDI

**Table 5:** Results from the logistic regression performed on risk of bleeding from 3867 patients treated with oral anticoagulant in primary care setting before their hospitalization. An OR > 1 was considered as a risk factor and a *p*-value < 0.05 was considered as significant. OR = Odds Ratio, CI = Confidence Interval.

Characteristic	OR	95% CI	*p*-Value
**Sex**			
*F*	-	-	-
*M*	1.19	1.00–1.43	0.053
**Age**	1.03	1.02–1.04	<0.001
**History of stroke**	1.17	0.64–2.03	0.60
**Previous bleeding event**	4.23	3.00–5.94	<0.001
**Diabetes**	1.29	0.99–1.66	0.058
**Renal disease**	1.98	1.48–2.64	<0.001
**Liver disease**	1.72	0.90–3.14	0.085
**Cancer**	2.68	1.56–4.50	<0.001
**Hypertension**	1.72	1.43–2.06	<0.001
**PIM**			
*0*	-	-	-
*1*	1.08	0.85–1.38	0.500
**DDI**			
*0*	-	-	-
*1*	1.15	0.92–1.45	0.200
**PIM–DDI**			
*0*	-	-	-
*1*	1.23	1.00–1.57	0.060

**Table 6:** Model performances. Accuracy (proportion of the data that are predicted correctly). Sensitivity (proportion of positive results out of the number of samples, which were actually positive). Specificity (proportion of negatives that are correctly identified as negatives). RF = Random Forest, XGBoost = Extreme Gradient Propulsion and SVM = Support Vector Machine.

	RF	XGBoost	SVM
**Accuracy**	0.64	0.68	0.64
**Sensitivity**	0.65	0.70	0.56
**Specificity**	0.64	0.68	0.65

## Data Availability

Not applicable.

## Supplementary Materials

The following supporting information can be downloaded at: https://www.mdpi.com/article/10.3390/pharmaceutics14071410/s1, Table S1: ICD-10 diagnoses codes used to detect bleeding ADE at hospital admission.
