# Supplementary material for: Drug–Drug Interactions with Oral Anticoagulants as Potentially Inappropriate Medications: Prevalence and Outcomes in Elderly Patients in Primary Care and Hospital Settings
Source: Pharmaceutics. 2022 Jul 5;14(7):1410. doi: 10.3390/pharmaceutics14071410 (PMC9325322; doi:10.3390/pharmaceutics14071410)
Supplement: Supplementary file 1 [file pharmaceutics-14-01410-s001.zip › pharmaceutics-1751054-supplementary.pdf]

Table S1: ICD-10 diagnoses codes used to detect bleeding ADE at hospital admission

	ICD10	Bleeding adverse event
Other bleedings	D50.0	Iron deficiency anaemia secondary to blood loss
	D62	Acute posthaemorrhagic anaemia
	H11.3	Conjunctival haemorrhage
	H21.0	Hyphaema
	H35.6	Retinal haemorrhage
	H43.1	Vitreous haemorrhage
	M25.0	Haemarthrosis
	R04.0	Epistaxis
	R23.3	Spontaneous ecchymoses
	R58	Haemorrhage, not elsewhere classified
	T45.5	Poisoning by, adverse effect of and underdosing of anticoagulants
	Y44.2	Adverse effects in therapeutic use: anticoagulants
Gastro intestinal bleedings	K06.8	Other specified disorders of gingiva and edentulous alveolar ridge
	K25.0	Gastric ulcer : acute with haemorrhage
	K25.2	Gastric ulcer : acute with both haemorrhage and perforation
	K25.4	Gastric ulcer : chronic or unspecified with haemorrhage
	K25.6	Gastric ulcer : chronic or unspecified with both haemorrhage and perforation
	K26.0	Duodenal ulcer : acute with haemorrhage
	K26.2	Duodenal ulcer : acute with both haemorrhage and perforation
	K26.4	Duodenal ulcer : chronic or unspecified with haemorrhage
	K26.6	Duodenal ulcer : chronic or unspecified with both haemorrhage and perforation
	K27.0	Peptic ulcer : acute with haemorrhage
	K27.2	Peptic ulcer : acute with both haemorrhage and perforation
	K27.4	Peptic ulcer : chronic or unspecified with haemorrhage
	K27.6	Peptic ulcer : chronic or unspecified with both haemorrhage and perforation
	K28.0	Gastrojejunal ulcer : acute with haemorrhage
	K28.2	Gastrojejunal ulcer : acute with both haemorrhage and perforation
	K28.4	Gastrojejunal ulcer : chronic or unspecified with haemorrhage
	K28.6	Gastrojejunal ulcer : chronic or unspecified with both haemorrhage and perforation

	K29.0	Acute haemorrhagic gastritis
	K92.2	Gastrointestinal haemorrhage unspecified
	K62.5	Haemorrhage of anus and rectum
	K66.1	Haemoperitoneum (excl. Traumatic haemoperitoneum)
	K92.0	Haematemesis
	K92.1	Melaena
	R04.1	Haemorrhage from throat
Intracranial bleedings	I60	Subarachnoid haemorrhage
	I60.0	Subarachnoid haemorrhage from carotid siphon and bifurcation
	I60.1	Subarachnoid haemorrhage from middle cerebral artery
	I60.2	Subarachnoid haemorrhage from anterior communicating artery
	I60.3	Subarachnoid haemorrhage from posterior communicating artery
	I60.4	Subarachnoid haemorrhage from basilar artery
	I60.5	Subarachnoid haemorrhage from vertebral artery
	I60.6	Subarachnoid haemorrhage from other intracranial arteries
	I60.7	Subarachnoid haemorrhage from intracranial artery, unspecified
	I60.8	Other subarachnoid haemorrhage
	I60.9	Subarachnoid haemorrhage, unspecified
	I61	Intracerebral haemorrhage
	I61.0	Intracerebral haemorrhage in hemisphere, subcortical
	I61.1	Intracerebral haemorrhage in hemisphere, cortical
	I61.2	Intracerebral haemorrhage in hemisphere, unspecified
	I61.3	Intracerebral haemorrhage in brain stem
	I61.4	Intracerebral haemorrhage in cerebellum
	I61.5	Intracerebral haemorrhage, intraventricular
	I61.6	Intracerebral haemorrhage, multiple localized
	I61.8	Other intracerebral haemorrhage
	I61.9	Intracerebral haemorrhage, unspecified
	I62	Other nontraumatic intracranial haemorrhage
	I62.0	Nontraumatic subdural haemorrhage
	I62.1	Nontraumatic extradural haemorrhage
	I62.9	Intracranial haemorrhage (nontraumatic), unspecified

Respiratory bleedings	R04.2	Haemoptysis
	R04.8	Haemorrhage from other sites in respiratory passages
	R04.9	Haemorrhage from respiratory passages, unspecified
Genitourinary bleedings	I31.2	Haemopericardium, not elsewhere classified
	N93.9	Abnormal uterine and vaginal bleeding, unspecified
	R31	Unspecified haematuria
